# The Effects of Spatial Attention Focus and Visual Awareness on the Processing of Fearful Faces: An ERP Study

**DOI:** 10.3390/brainsci12070823

**Published:** 2022-06-24

**Authors:** Zeguo Qiu, Stefanie I. Becker, Alan J. Pegna

**Affiliations:** School of Psychology, The University of Queensland, Brisbane 4072, Australia; zeguo.qiu@uq.net.au (Z.Q.); s.becker@psy.uq.edu.au (S.I.B.)

**Keywords:** fearful face, spatial attention, awareness, emotion processing, ERP

## Abstract

Previous research on the relationship between attention and emotion processing have focused essentially on consciously-viewed, supraliminal stimuli, while the attention-emotion interplay remains unexplored in situations where visual awareness is restricted. Here, we presented participants with face pairs in a backward masking paradigm and examined the electrophysiological activity in response to fearful and neutral expressions under different conditions of attention (spatially attended vs. unattended) and stimulus visibility (subliminal vs. supraliminal). We found an enhanced N2 (visual awareness negativity -VAN-) and an enhanced P3 for supraliminal compared to subliminal faces. The VAN, indexing the early perceptual awareness, was enhanced when the faces were spatially attended compared to when they were unattended, showing that the VAN does not require spatial attention focus but can be enhanced by it. Fearful relative to neutral expressions enhanced the early neural activity (N2) regardless of spatial attention but only in the supraliminal viewing condition. However, fear-related enhancements on later neural activity (P3) were found when stimuli were both attended and presented supraliminally. These findings suggest that visual awareness is needed for emotion processing during both early and late stages. Spatial attention is required for emotion processing at the later stage but not at the early stage.

## 1. Introduction

### 1.1. The Role of Spatial Attention Focus in Emotion Processing

Emotional faces constitute important social information in our daily life. Expressions like fear can act as cues for potential threats in the environment and are therefore thought to be prioritised for attention. The interplay between attention and emotion processing has been extensively investigated using emotional faces. Especially for negative expressions, previous studies have shown that they compete more for attentional resources, compared to neutral faces [1,2,3]. One aspect of the attentional bias to emotional faces is their access to conscious awareness independent of spatial attention focus. Indeed, it has been suggested that emotional faces can be processed pre-attentively, i.e., without spatial attention focus [4]. However, other researchers argue that emotion processing requires attentional focus [5,6].

The question of whether emotional faces can be processed outside the focus of attention has been investigated using electroencephalography or EEG, a useful tool for revealing the electrical activity during a wide range of cognitive processes in the human brain. Using EEG, it has been shown that emotional compared to neutral faces can increase the amplitudes of event-related potentials (ERPs) from mid-latency onwards (i.e., N170, N2, P3 [2]. Specifically, the P3, an ERP component with an onset of around 300 ms post-stimulus at parietal regions, can be most consistently increased for emotional relative to neutral faces when attention is directed to the facial expressions of the stimuli [2]. It is possible that later stages of visual processing for emotional faces, as characterised by the P3, need attention.

In comparison, emotional expressions, in particular fear and anger, produce an enhancement effect on earlier ERPs (i.e., N170, N2) largely independently of whether they are attended or not, across different attention tasks [2]. For example, Huang and colleagues found that, unattended fearful faces presented laterally could enhance an early component (i.e., P2), compared to unattended neutral faces, but this effect was modulated by participants’ attentional load [7]. Similarly, using affective non-face pictures, early and mid-latency components (i.e., N1, P2, N2) were enhanced for unattended negative-valence pictures, compared to unattended neutral pictures, only in a low attentional load condition [8,9]. These results showed that unattended emotional stimuli can enhance neural activity under certain circumstances. However, it is unclear how the emotion-related modulations on the ERPs compare directly to situations where the faces are attended.

Moreover, other studies found that emotional faces did not enhance early ERPs when they were not spatially attended. In one previous study, participants were presented with a vertical/horizontal pair of house images and a horizontal/vertical pair of face images and had to attend to either the horizontal or vertical pair of stimuli [6]. It was found that, when the faces were spatially attended, the fearful expression of the faces enhanced an early frontal positivity starting at around 100 ms post-stimulus, compared to neutral faces. When the faces were unattended, however, no emotion-related effect could be found on the ERPs [6]. Correspondingly, the authors concluded that spatial attention gates emotion processing even at an early stage of processing.

Similarly, in another ERP study, participants were presented with pairs of lateral face images and were asked to either discriminate an emotional face from a neutral one, or compare the lengths of two lines presented close to the screen centre [5]. The fearful expression was found to enhance the early frontal positivity and the N2 at posterior electrodes, when participants had to indicate the emotional expressions of the faces. However, the emotion-related effects on the ERPs disappeared when spatial attention was directed away from the faces and engaged in the rather demanding line task [5]. It was concluded that, again, the processing of emotional expressions requires spatial attention.

Therefore, it is still disputed to date whether emotion processing can indeed occur outside spatial attention focus. To address this question, it would be necessary to perform direct comparisons between responses to attended and unattended faces while assessing the neural markers for emotional face processing across different attentional conditions. Specifically, does the strength of neural activity differ for emotional and neutral expressions for an unattended face? How does the effect compare to the emotion-related modulations on an attended face?

### 1.2. The Role of Awareness in Emotion Processing

Note that the ERP studies described above all allowed supraliminal viewings of the stimuli. The findings on the attention-emotion interplay should therefore be interpreted in the context of conscious emotion processing. In other words, while emotional expressions may be processed when the faces are outside spatial attention focus, it is unknown whether this effect depends on participants’ awareness of the stimuli.

Previous research has shown that early ERP components like the face-sensitive N170 can be enhanced by emotional expressions in both supraliminal and subliminal viewing conditions [10,11,12]. The enhanced N170 for emotional faces presented subliminally has been taken as evidence that emotional expressions can be processed without visual awareness. However, in these studies, faces were often presented at the centre of the screen and the implementation of inattention to the faces was rare. Specifically, it has not yet been examined whether any nonconscious emotion processing can occur outside the focus of spatial attention. Relevant to this particular question, one previous ERP study examined the relationship between visual awareness and emotion processing for faces that were irrelevant to the experimental task [11]. In their study, participants were presented with a central face stimulus, backward masked, either subliminally (16 ms) or supraliminally (166 ms) and were asked to compare the lengths of two vertical lines presented on either side of the face [11]. It was found that the fearful expression of the task-irrelevant faces enhanced the N170, compared to a neutral expression, regardless of stimulus visibility [11]. The authors concluded that subliminal processing of facial expressions is possible and that it can occur outside participants’ attentional focus [11].

However, because the face stimuli were presented at the screen centre where participants’ overt attention was focused, it is questionable whether the faces indeed remained unattended. Instead, their implementation of inattention was mitigated by task-relevancy of the faces. Therefore, while this previous study provided some support for nonconscious processing of emotional expressions of task-irrelevant faces, it is difficult to conclude unequivocally that the stimuli were processed outside the focus of spatial attention. It thus remains an open question whether nonconscious processing of emotion is independent of spatial attention.

In addition, the subliminal emotion-related effect on the N170 has been found to occur prior to the emergence of the visual awareness negativity or the VAN [10,12], an indicator of early perceptual awareness. Studies on awareness would benefit from an examination of the awareness-related components, such as the VAN, as they provide information about whether and how the neural correlates of visual awareness can be modulated by experimental manipulations.

The VAN is a relative negativity in ERP signals appearing at 200–300 ms post-stimulus for supraliminal compared to subliminal stimuli over occipito-temporal electrodes, and it has been suggested to index an early, perceptual stage of awareness [13]. Another potential neural correlate of awareness is the P3, a positive-going wave appearing at around 300–600 ms post stimulus at parietal regions, which is also greater for consciously perceived stimuli compared to unconscious stimuli [14]. The P3 has been suggested to index a later, reflective stage of awareness [13,15]. Additionally, as a relatively later component in visual processing, the P3 has been linked to a variety of awareness-unrelated cognitive processes [16,17], whereas the VAN is suggested to be the earliest component related to visual awareness in the human brain [18]. Thus far, there has been very limited evidence on whether these awareness-related components, namely the VAN and the P3, can be modulated by the emotional valence of face stimuli.

Similarly, the investigation on the relationship between awareness-related components, the VAN in particular, and spatial attention is lacking. Several studies showed that the N2-posterior-contralateral (the N2pc), the neural marker for spatial attention shifting, could be enhanced with higher levels of awareness [19,20] or was present only when participants were aware of the stimuli [21,22]. However, the examination of how the neural markers for awareness (i.e., the VAN and the P3) can in turn be modulated by attention is limited yet indispensable to a comprehensive understanding of the attention-awareness relationship. In a previous VAN study, Koivisto and colleagues used a bilateral presentation of letters in conjunction with backward masking to investigate the interactions between the VAN and spatial attention [23]. It was found that successful detection of a target letter and the VAN were dependent on the focus of spatial attention. Specifically, the VAN was only observed in the spatial visual field participants selectively attended but not in the unattended visual field [23]. It has not yet been studied, however, whether this pattern can be observed for more complex and biologically meaningful stimuli such as human faces.

Therefore, to better understand the relationship between visual awareness, spatial attention focus and emotion processing, we incorporated both inattention and unawareness in the present experiment. Importantly, we made the emotion of face stimuli task-relevant by asking participants to respond to the emotion of the face appearing on the attended location while ignoring the face on the unattended location. Meanwhile, to allow an examination of visual awareness, we manipulated stimulus visibility (i.e., supraliminal vs. subliminal) by using backward masking, a technique shown to be efficient at suppressing awareness [24,25]. We primarily focused on the awareness-related components (i.e., the VAN and the P3) as well as the N170 and assessed how they could be modulated by attention to and the emotion of the presented faces.

We first predicted that, if spatial attention is necessary for visual awareness of faces to arise, we should observe the VAN only for the attended faces and not for the unattended faces. However, if visual awareness of faces does not depend on spatial attention, the VAN should be found in both spatially attended and unattended conditions. Second, if spatial attention focus is necessary for the processing of emotional expressions, the ERPs should be enhanced by fearful compared to neutral expressions only for the spatially attended faces. However, if emotion processing is independent of spatial attention focus, such enhancements should be found in both attended and unattended conditions. Furthermore, we aimed to determine whether any modulatory effects of spatial attention focus on emotion processing can be observed in both subliminal and supraliminal viewing conditions.

## 2. Materials and Method

### 2.1. Participants

We determined the sample size based on a previously reported effect of attention on the N2 (*η_p_*^2^ = 0.36) [10]. For our repeated-measures ANOVAs, in order to obtain a significant main effect of attention with a power of 90% and an effect size of 0.36 (alpha level = 0.05, two tailed), 22 participants were required (calculated with MorePower Software) [26]. We recruited 23 individuals with normal or corrected-to-normal vision at the University of Queensland. Participants reported no history of psychiatric or neurological conditions and each received 40 Australian dollars for their participation. We excluded data from one participant from further analyses after pre-processing the data (see EEG recording and pre-processing). Therefore, the final sample size was 22 (*M_age_* = 23 years, *SD_age_* = 4 years; 6 males, 16 females; 21 right-handed). The experimental procedure was approved by the ethics committee of University of Queensland. All participants provided informed consent prior to their participation.

### 2.2. Apparatus and Stimuli

We presented all experimental stimuli on a 24-inch ASUS LCD monitor model VG248QE (resolution: 1920 × 1080 pixels; refresh rate: 144 Hz). The distance between participant’s eyes and the monitor was 70 cm. An open software PsychoPy3 [27] was used to present stimuli and record participants’ behavioural responses.

**Figure 1:**
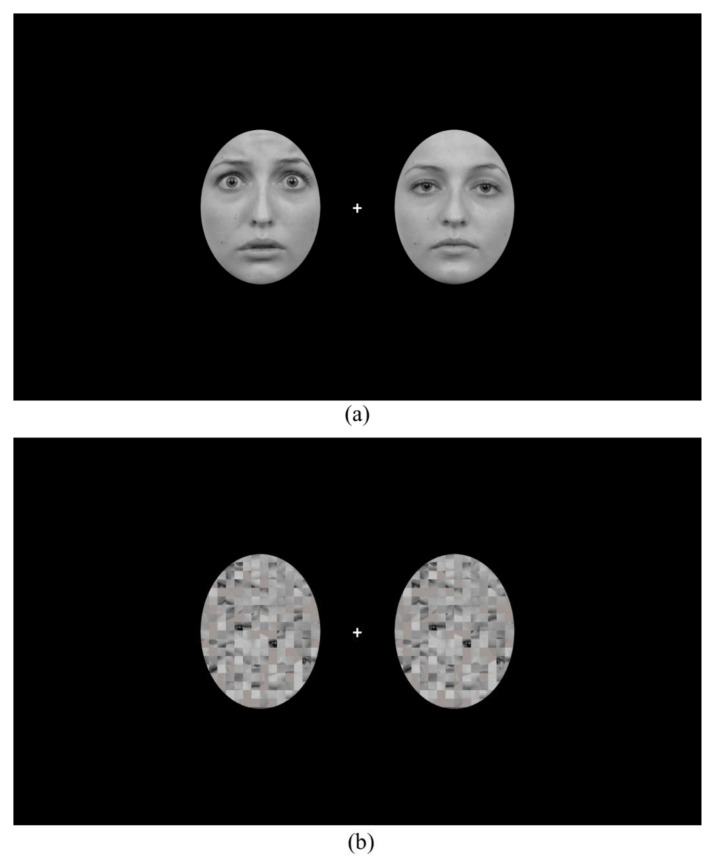
An example of (**a**) the face presentation (fearful face on the **left** and neutral face on the **right**) and an example of (**b**) the mask presentation.

Fearful and neutral face images of 16 different models (8 males, 8 females) were obtained from the Karolinska Directed Emotional Faces Database [28]. All images were rendered black-and-white and were presented on a black screen. As shown in Figure 1a, face images were cropped into an oval shape of 6.5° × 5.1° (in visual angle) so that non-facial information including hair was removed for each image. To generate mask stimuli, we used the Scramble Filter tool (http://telegraphics.com.au/sw/product/scramble; accessed on 31 January 2021) on neutral faces, which produced scrambled images where the face was unidentifiable while the overall image luminance remained the same (Figure 1b). For each face (or mask) presentation, two face (or mask) images from a same model were presented bilaterally with the centre of the image pair positioned 4.1° away from a central fixation cross on the screen. There were four combinations of face images: (a) two fearful faces; (b) fearful face on the left and neutral face on the right; (c) neutral face on the left and fearful face on the right; (d) two neutral faces.

### 2.3. Procedure

**Figure 2:**
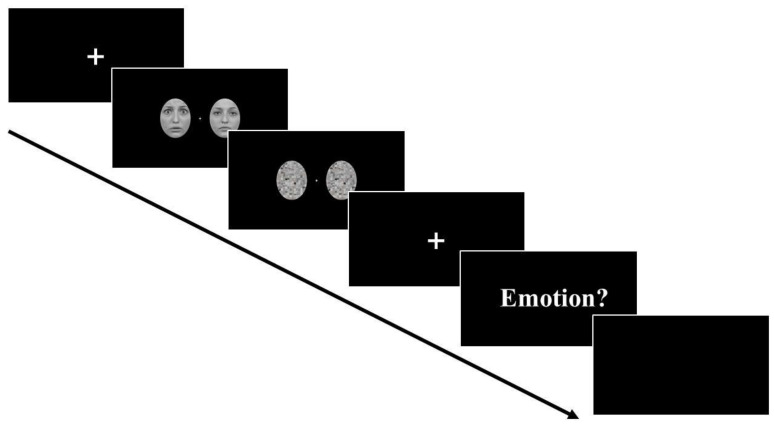
Time-course of events during a trial of the full experimental procedure.

Each trial started with a fixation screen (presented for a variable duration between 500 to 800 ms), followed by a pair of face images that could be one of the four combinations mentioned above (see Figure 2). The faces were presented for either 16 ms (subliminal) or 266 ms (supraliminal) and immediately followed by a pair of mask images that was presented for either 324 ms or 74 ms. As a result, the total duration of the face and mask stimuli was 340 ms for all conditions. Then, a fixation screen of 550 ms appeared and the participants were asked to respond.

At the beginning of the blocks, participants were instructed both verbally and with written instructions on the screen to covertly attend to one side of the screen while keeping their eyes fixated at the central fixation cross, and use the up and down arrow keys to report the emotion of the face presented at the attended side (e.g., up arrow key = fearful, down arrow key = neutral). A blank screen of 1000 ms was presented before the next trial began. Participants attended to either the left or right side of the screen for the first half of the experiment and attended to the opposite side for the second half of the experiment. The response button assignment was counterbalanced across participants.

Participants were instructed to respond as accurately as possible after the question cue appeared on the screen (as a result, we did not examine reaction time data). There were eight blocks of 96 trials in total with short breaks provided between blocks. Each of the four face combinations was presented 192 times in total, randomly intermixed within each block.

### 2.4. EEG Recording and Pre-Processing

EEG was recorded using the BioSemi ActiveTwo 64-electrodes system (sampling rate: 1024 Hz; Biosemi, Amsterdam, The Netherlands). Electrodes were applied according to the extended international 10–20 system location. Signals were referenced online to the CMS/DRL electrodes. An external electrode was placed below participants’ left eye and used in combination with FP1 to record vertical electrooculogram (EOG). Horizontal EOG was recorded using a pair of bipolar electrodes.

All steps of EEG data pre-processing were performed with EEGLAB [29] and ERPLAB [30]. Individual electrodes that produced sustained noise throughout the experiment were interpolated for the whole dataset. Signals were re-sampled to 512 Hz offline, filtered from 0.1 to 30 Hz and re-referenced to the average of all electrodes. Line noise was removed with a notch filter of 50 Hz. ERP signals were averaged and segmented into epochs with a time window of 0–600 ms, time-locked at the onset of the face images, and were baseline-corrected using a pre-stimulus baseline (−100 to 0 ms). We detected and removed trials with ocular artefacts (i.e., eye blinks and eye movements) semi-automatically on a trial-by-trial basis, using a threshold of −100 to 100 µV. Trials with other artefacts were detected and removed semi-automatically with a threshold of −80 to 80 µV. After artefact rejection, data from one participant were excluded from further analyses due to a low number of epochs left (i.e., fewer than 40 epochs in one of the conditions). On average, 85% epochs were kept for the remaining participants.

### 2.5. ERP Data Analysis

Although common brain regions have been reported to be linked to the VAN and the P3, there have been some inconsistencies in the electrode sites where these ERPs can be found [13], especially for complex stimuli like human faces [2]. Thus, we took a data-driven approach to identify the electrodes and time windows for the ERPs of interest by performing a Mass Univariate Analysis (MUA). The MUA was performed over all time-points within the ERP epochs (i.e., 0–600 ms) and all electrodes for significant differences (two-tailed α = 0.05) using a cluster-based permutation test (2500 permutations) to control for multiple comparisons [31]. With the cluster formation threshold set at 0.05, an electrode was considered as spatial neighbour to another if the distance between the two electrodes was within approximately 3.9 cm. As a result, each electrode had 3.7 spatial neighbours on average [31]. The MUA was performed using the Mass Univariate ERP Toolbox (https://openwetware.org/wiki/Mass_Univariate_ERP_Toolbox; accessed on 1 February 2022).

#### 2.5.1. VAN and P3

**Figure 3:**
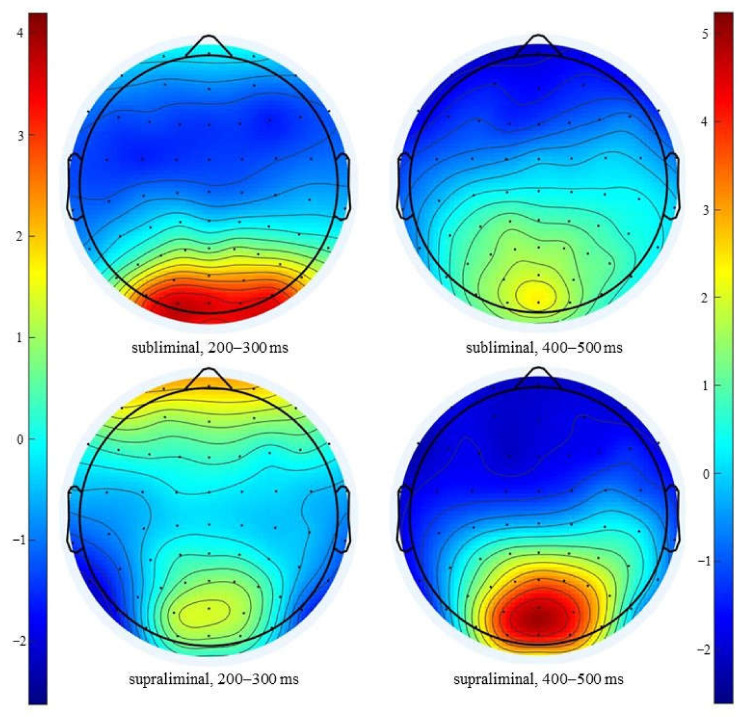
Topographic maps for the mean amplitudes in subliminal and supraliminal conditions, collapsed across face combinations, in the VAN time window (200–300 ms; **left** panel) and the P3 time window (400–500 ms; **right** panel).

For the VAN and the P3, electrodes and time windows were identified as those that showed a significant difference between the supraliminal and subliminal conditions. Specifically, we first obtained the average bins separately for supraliminal conditions and subliminal conditions, across all face combinations. Then we calculated the difference bin by subtracting the average subliminal bin from the average supraliminal bin. The MUA was performed on the difference bin. Topographic maps for subliminal and supraliminal conditions at the VAN and P3 time windows are shown in Figure 3.

A significant effect of stimulus visibility was found on electrodes TP7/8, P5/6, P7/8, P9/10, O1/2, PO3/4, PO7/8, POz, Oz, Iz in a common time window of 200–300 ms, consistent with a VAN. In order to examine any effect of spatial attention focus on the ERP signals in this time window, we included the laterality based on the attended face (contralateral vs. ipsilateral signals to the attended face) as a variable for our analysis on the VAN data. As a result, we used the lateral electrodes for the VAN (TP7/8, P5/6, P7/8, P9/10, O1/2, PO3/4, PO7/8) and exported the mean amplitudes between 200 and 300 ms from these electrodes, separately for the left and right hemispheres.

A significant effect of stimulus visibility was also found on electrodes Pz, POz, Oz, P1/2, P3/4, PO3/4, O1/2 in a common time window of 400–500 ms, reflecting an enhanced positivity for supraliminal stimuli on these electrodes. We thus pooled data from these electrodes and exported the mean signal amplitudes of the specified time window for the analysis of the P3.

#### 2.5.2. N170

The N170 has been shown to be enhanced by subliminally presented emotional faces in previous literature [10,11,12]. Therefore, to examine any nonconscious processing of emotion, we additionally analysed the mean amplitudes of the N170 time window. In keeping with our electrode and time window selection strategy, a MUA was performed on the subliminal condition, averaged across all face combinations. A significant negativity was found in a common time window of 130–190 ms over electrodes TP7/8, P3/4, P5/6, P7/8, P9/10, PO7/8. The mean signal amplitudes were pooled over these electrodes between 130 and 190 ms, separately for the left and right hemispheres, for the N170 analysis.

All statistical analyses were performed in IBM SPSS Statistics 27. The *p* values for post-hoc comparisons were adjusted using the Holm-Bonferroni correction method.

## 3. Results

### 3.1. Behavioural Results

Participants’ accuracy at the emotion detection task was submitted to a 2(stimulus visibility: subliminal, supraliminal) × 2(emotion of the attended face: fearful, neutral) × 2(emotion of the unattended face: fearful, neutral) repeated-measures ANOVA. A main effect of stimulus visibility was found, *F*(1, 21) = 1931.73, *p* < 0.001, *η_p_*^2^ = 0.99, whereby participants were more accurate in the supraliminal condition (*M* = 0.92, *SD* = 0.04) than in the subliminal condition (*M* = 0.52, *SD* = 0.02). The main effect of the emotion of the attended face was also significant, *F*(1, 21) = 23.49, *p* < 0.001, *η_p_*^2^ = 0.53, such that neutral faces (*M* = 0.82, *SD* = 0.10) were more accurately detected, compared to fearful faces (*M* = 0.62, *SD* = 0.10). However, this effect was modulated by stimulus visibility, *F*(1, 21) = 17.52, *p* < 0.001, *η_p_*^2^ = 0.46. Follow-up *t*-tests showed that the attended neutral faces were more accurately detected than the attended fearful faces only when the stimuli were presented subliminally, *t*(21) = 4.71, *p* < 0.001, *d* = 1.00, not when they were presented supraliminally, *t*(21) = 1.51, *p* = 0.146.

We additionally derived d-prime (d′) and criterion (c) from signal detection theory [32,33] to examine discriminability of the targets and any bias in their responses, respectively. Specifically, the number of hits and false alarms for fearful faces were calculated for each participant. Since d′ is an open-ended scale, values vary from 0 (guessing) to values typically of 2 and above (representing good discriminability of targets). A c of a value of 0 reflects no bias in the responses. Because we calculated the hits and false alarms for fearful faces, a negative c value indicates a bias to a fearful face response whereas a positive value indicates a bias to a neutral face response.

Consistent with the accuracy results, participants had chance-level discriminability of emotion in the subliminal condition (d′ = 0.07; *SD* = 0.10) but showed very good discrimination performance in the supraliminal condition (d′ = 2.42; *SD* = 0.87). The criterion results show that, at the subliminal level, participants showed a bias to a neutral face response (c = 1.10, *SD* = 2.18). However, at the supraliminal level, there seemed to be no bias in participants’ responses (c = 0.27, *SD* = 0.80). Therefore, the overall more accurate detection of neutral faces in the subliminal condition was likely driven by a response bias towards the neutral face response.

### 3.2. ERP Amplitudes

#### 3.2.1. VAN Time Window (200–300 ms)

**Figure 4:**
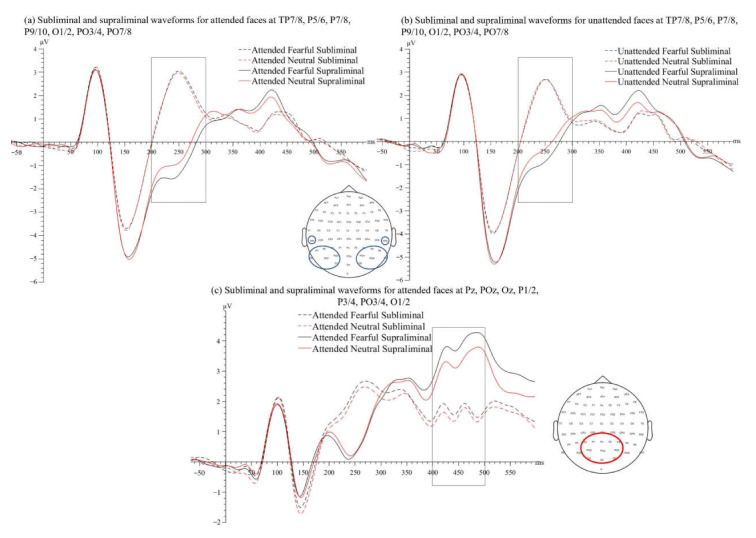
ERP waveforms for (**a**) attended and (**b**) unattended faces in subliminal and supraliminal viewing conditions, separated by the emotional expression (fearful and neutral), pooled from **left** (TP7, P5, P7, P9, O1, PO3, PO7) and **right** electrodes (TP8, P6, P8, P10, O2, PO4, PO8) for the VAN (time window: 200–300 ms). (**c**) ERP waveforms for attended fearful and neutral faces, averaged across the emotion of the unattended faces in subliminal and supraliminal viewing conditions, pooled from Pz, POz, Oz, P1/2, P3/4, PO3/4, O1/2 for the P3 (time window: 400–500 ms).

A 2(stimulus visibility: subliminal, supraliminal) × 2(laterality of the attended face as referred to electrodes: contralateral, ipsilateral) × 2(emotion of the attended face: fearful, neutral) × 2(emotion of the unattended face: fearful, neutral) repeated-measures ANOVA was performed on the mean ERP amplitudes of the VAN time window. The main effect of stimulus visibility was significant, *F*(1, 21) = 36.47, *p* < 0.001, *η_p_*^2^ = 0.64, with more negative ERPs in the supraliminal condition (*M* = −0.60 µV, *SD* = 1.98) than the subliminal one (*M* = 1.79 µV, *SD* = 1.77), consistent with the VAN, see Figure 4a,b.

The interaction between stimulus visibility and laterality was significant, *F*(1, 21) = 17.36, *p* < 0.001, *η_p_*^2^ = 0.45. Follow-up *t*-tests showed that the effect of laterality was non-significant at the supraliminal level, *t′*(21) = 1.57, *p_corr_* = 0.264, or at the subliminal level, *t′*(21) = 2.06, *p_corr_* = 0.157. Therefore, an N2pc effect [34,35] was not observed in the selected VAN time window. The significance of the visibility-by-laterality interaction was reflected in a larger supraliminal-subliminal difference (i.e., VAN) in the contralateral relative to the ipsilateral condition. A paired-samples *t*-test showed that the VAN contralateral to the attended face (*M* = −2.71, *SD* = 2.09) was larger than the VAN ipsilateral to the attended face (*M* = −2.07, *SD* = 1.67), *t*(21) = 4.17, *p* < 0.001, *d* = 0.89, suggesting that the preparatory focus of spatial attention enhanced perceptual awareness indexed by the VAN.

The interaction between stimulus visibility and the emotion of the attended face was also significant, *F*(1, 21) = 11.47, *p* = 0.003, *η_p_*^2^ = 0.35. Follow-up *t*-tests showed that, at the subliminal level, the amplitudes of ERPs for fearful and neutral faces did not differ, *t′*(21) = 1.07, *p_corr_* = 0.297. However, at the supraliminal level, ERP signals associated with the attended fearful faces (*M* = −0.77 µV, *SD* = 1.94) were more negative than the attended neutral faces (*M* = −0.43 µV, *SD* = 2.05), *t′*(21) = 3.58, *p_corr_* = 0.007, *d′* = 0.76.

The main effect of the emotion of the unattended face was significant, *F*(1, 21) = 9.64, *p* = 0.005, *η_p_*^2^ = 0.32, with an unattended fearful face (*M* = 0.49 µV, *SD* = 1.61) showing less positive ERP signals in the time window, compared to an unattended neutral face (*M* = 0.70 µV, *SD* = 1.68). Our planned comparisons (paired-samples *t*-tests) showed that the ERP amplitudes did not differ between fearful and neutral expressions when the faces were not spatially attended and not consciously processed (i.e., subliminal condition), *t*(21) = 1.13, *p* = 0.271. However, compared to the neutral expression (*M* = −0.45 µV, *SD* = 2.02), the fearful expression of an unattended face (*M* = −0.75 µV, *SD* = 1.97) enhanced the ERPs when the faces were presented supraliminally, *t*(21) = 2.71, *p* = 0.013, *d* = 0.58. To directly compare the emotion-related effects between different spatial attention conditions, we computed the fearful-neutral differences for both attended and unattended faces in the supraliminal viewing condition. A paired-samples *t*-test showed that the emotion-related differences did not differ between attended and unattended conditions, *t*(21) = 0.35, *p* = 0.728.

No other effect was significant, *Fs* < 2.67, *ps* > 0.117.

To confirm that the above effects were not caused by potential overt attention towards the target screen side in the participants, we used data from the EOG channels to examine potential micro-saccadic movements in the attend-to-left and attend-to-right conditions, separately. Specifically, we calculated the horizontal EOG signals by subtracting the mean signals evoked by the face stimuli on the right EOG from those on the left EOG. One-sample *t*-tests showed that there was no shift in the EOG signals in either the attend-to-left condition, *t*(21) = 1.96, *p* = 0.064, or the attend-to-right condition, *t*(21) = 0.87, *p* = 0.394. Correspondingly, Bayesian one-sample *t*-tests provided anecdotal (BF_01_ = 1.12) and moderate evidence (BF_01_ = 4.27) for the null hypothesis, respectively. Therefore, the effects reported above were not due to micro-saccades to the target.

#### 3.2.2. P3 Time Window (400–500 ms)

A 2(stimulus visibility: subliminal, supraliminal) × 2(emotion of the attended face: fearful, neutral) × 2(emotion of the unattended face: fearful, neutral) repeated-measures ANOVA was performed on the mean ERP amplitudes of the P3 time window. The main effect of stimulus visibility was significant, *F*(1, 21) = 50.55, *p* < 0.001, *η_p_*^2^ = 0.71, whereby the ERPs in the supraliminal condition (*M* = 3.58 µV, *SD* = 2.47) were more positive than in the subliminal condition (*M* = 1.60 µV, *SD* = 1.87). We also found a main effect of the emotion of the attended face, *F*(1, 21) = 8.39, *p* = 0.009, *η_p_*^2^ = 0.29, reflecting that attended fearful faces (*M* = 2.76 µV, *SD* = 2.04) were associated with more positive ERPs, compared to attended neutral faces (*M* = 2.41 µV, *SD* = 2.18), see Figure 4c.

The main effect of the emotion of the unattended face was non-significant, *F*(1, 21) = 1.81, *p* = 0.193. No other effect was significant, *Fs* < 1.50, *ps* > 0.235. Thus, the P3 was modulated by stimulus visibility and emotion of the attended face, but not by the emotion of the unattended face.

#### 3.2.3. N170 Time Window (130–190 ms)

A 2(stimulus visibility: subliminal, supraliminal) × 2(laterality of the attended face: contralateral, ipsilateral) × 2(emotion of the attended face: fearful, neutral) × 2(emotion of the unattended face: fearful, neutral) repeated-measures ANOVA was performed on the mean ERP amplitudes of the N170 time window. The main effect of stimulus visibility was significant, *F*(1, 21) = 55.95, *p* < 0.001, *η_p_*^2^ = 0.73, whereby the N170 was larger in the supraliminal condition (*M* = −4.57 µV, *SD* = 2.47) than in the subliminal condition (*M* = −3.15 µV, *SD* = 2.11). No other effect was significant, *Fs* < 3.93, *ps* > 0.061.

To specifically examine any effect of emotion (fearful and neutral) on the N170, we compared the fearful-fearful faces condition against the neutral-neutral faces condition at both levels of stimulus visibility in a 2(stimulus visibility: subliminal, supraliminal) × 2(emotion: both fearful, both neutral) repeated-measures ANOVA. The effect of emotion was non-significant, *F* < 1, *p* = 0.874, as was the interaction between stimulus visibility and emotion, *F* < 1, *p* = 0.839. Thus, the N170 was only modulated by stimulus visibility.

## 4. Discussion

In this study, we examined the EEG activity in response to fearful and neutral human faces under different conditions of attention (spatially attended vs. spatially unattended) and awareness (subliminal vs. supraliminal). ERP signals between 200 and 300 ms and between 400 and 500 ms were larger for supraliminally presented faces than for subliminally presented ones, reflecting an enhanced N2 (i.e., the VAN) and an enhanced P3, respectively, for supraliminal faces. Regardless of the expressions, the VAN was enhanced when the faces were spatially attended compared to when they were unattended. The N2 was enhanced by fearful relative to neutral expressions regardless of spatial attention focus, but only in the supraliminal viewing condition. In comparison, the fear-related enhancements on the P3 required both spatial attention and awareness.

### 4.1. VAN Does Not Require Spatial Attention Focus but Can Be Enhanced by It

From the Mass Univariate Analysis, we found a significant difference between supraliminal and subliminal conditions in the time window of 200–300 ms over multiple posterior electrodes. Specifically, ERP signals were more negative in this time window for supraliminal relative to subliminal stimuli, consistent with the VAN, an indicator of perceptual awareness [13]. The literature has provided substantial support for the correlation between the VAN and visual awareness, with studies using a variety of experimental paradigms [13,15,36]. Specifically, after controlling for potential confounds including task performance [37] and task relevance of the stimuli [38], ERP amplitudes at around 200 ms were found to be more negative in trials where participants reported high levels of awareness of the visual stimuli, relative to trials where low levels of awareness or no awareness was reported.

In a later time window (400–500 ms), we found a larger positivity in the supraliminal condition than the subliminal condition, reflecting a stronger P3 for supraliminal stimuli. The P3 has been suggested to constitute another neural correlate of awareness and is proposed to index a later, reflective stage [15,39]. However, it has also been suggested that the P3 may not reflect awareness *per se*. Rather, it is characterised by a variety of post-perceptual processes including the evaluative appraisal of stimuli [13,38,40].

According to the *recurrent processing framework* [41,42], awareness arises as a result of feedforward activation of visual areas (e.g., V1) and recurrent activity that takes place both within the activated areas and across the cortices (e.g., from higher areas to V1). By presenting backward masks immediately after stimuli that are presented briefly, the recurrent activity that is necessary for awareness may be hindered, leading to reduced neural activity, compared to when the same information is presented supraliminally. The VAN has been suggested to index the ERP differences between supraliminal and subliminal conditions [43]. Here, we show that complex and meaningful stimuli like human faces are associated with a large VAN when they are clearly visible to the participants and gain access to awareness, compared to when they are rendered subliminal and hence not consciously processed, consistent with previous studies also using face stimuli [22,44,45].

Furthermore, the recurrent processing model also posits that attention enables recurrent neural activity on a larger scale, which renders a comprehensive processing of visual information possible [41,42]. Consistent with this, we found that the VAN was larger when the faces were spatially attended. However, the VAN was also present for the unattended faces, showing that spatial attention is not necessary for obtaining a significant VAN.

This finding is in line with previous studies showing that visual awareness is independent from spatial attention [46,47,48,49,50]. For example, in a study using peripheral cues to manipulate spatial attention [47], neural activity associated with participants’ awareness of a change of the stimulus (i.e., the VAN) was comparable between trials where the changed stimulus was spatially cued and trials where it was not cued. It was therefore suggested that the VAN was independent of spatial attention. Similarly, using a spatial cueing task in conjunction with the magnetoencephalography, Wyart and Tallon-Baudry found that spatial attention and perceptual awareness were associated with separable neural oscillatory activity patterns [50]. In agreement with these findings, we found the VAN in both conditions of spatial attention (attended vs. unattended). Further to this, our results revealed that the VAN was larger when the faces were presented at the attended location, showing that spatial attention focus enhanced perceptual awareness of the faces, in line with the recurrent processing model of awareness [41,42].

Some may argue that the increase in the VAN for the attended relative to unattended faces is due to an effect of the N2pc, an indicator of spatial attention shifting [34,35], towards the attended side in our study. However, we did not find a corresponding N2pc. Moreover, the N2pc has been shown to index the shift of spatial attention, rather than the preparatory focus of spatial attention [51,52,53]; but see [54]. Therefore, we argue that the larger VAN for an attended face was not driven by spatial attention shifting towards it. Rather, it reflected enhanced perceptual awareness of the face because it was presented in an attended spatial region.

While our findings are in line with the view that attention and awareness are at least partly independent, some researchers oppose this by suggesting that visual awareness cannot occur without attention [55]. The discrepancies between our current findings and certain reports in the literature may be partly reconciled by considering the multifaceted nature of attention. Specifically, in the current study, we found that perceptual awareness of fearful faces (the VAN) was independent of, but still modulated by spatial attention focus. However, other forms of attention, for example, spatial attention shifting [22] and feature-based attention [23], may interact differently with perceptual awareness, or specifically the VAN. Future studies should thus seek to distinguish different forms of attention when examining their relationships with visual awareness.

### 4.2. Early Emotion Processing Needs Awareness but Not Spatial Attention

Our next questions were whether the processing of emotional expressions depended on spatial attention focus and whether this attention-emotion relationship could be affected by visual awareness. We found that ERPs between 200 and 300 ms (N2) were enhanced by fearful expressions, compared to neutral ones, but only in the supraliminal viewing condition. However, this conscious emotion-related modulation was found in both spatially attended and unattended conditions. Therefore, the processing of the fearful expression required visual awareness but not spatial attention focus at the early stage of processing (i.e., 200–300 ms).

This finding is at odds with previous studies where spatial attention focus was found to be necessary for processing the fearful expression of visible faces [5,56]. However, methodological concerns may restrict the interpretability of some of the previous results. For example, in the study by Eimer and collaborators, participants were required to explicitly evaluate the emotional expressions in the face-attended condition whereas, in the face-unattended condition, the faces were made completely task-irrelevant [5]. As a result, while differences in the emotion-related effects between attended and unattended conditions could be due to spatial attention, they may also reflect the effects of task-relevancy of the faces [5]. Specifically, during the line task, task-irrelevant faces may be suppressed to allow an accurate comparison of the task-relevant lines, potentially resulting in the complete elimination of emotion-related modulations on the ERPs. Therefore, the extent to which the findings indeed informed the relationship between spatial attention and emotion processing *per se* was not clear.

Here, we removed the confounding effects of task-relevancy between spatially attended and unattended conditions by asking the participants to evaluate the emotion of the attended faces explicitly. With these implementations, we found that, when consciously processed, fearful expressions enhanced the N2 both when the faces were spatially attended and unattended, and that the emotion effects were comparable between the attended and unattended conditions.

Furthermore, we did not find any emotion-related effects on the ERPs in the subliminal viewing condition, in contrast with previous research where fearful expressions were found to be processed in the absence of awareness [10,11,12]. In these previous studies, centrally presented fearful faces enhanced the N170 relative to neutral faces in subliminal viewing conditions [10,12]. However, in the current study, the N170 amplitudes did not differ between fearful and neutral faces, even when the faces were presented supraliminally. In our paradigm, two faces were presented bilaterally and the participants had to covertly attend to a lateralised face in the pairs. Perhaps, the processing of a lateralised stimulus using covert attention is not as efficient as the processing of a stimulus presented at the centre of visual fields [11]. Also, it is possible that the two lateralised faces competed for neural representation. Specifically, when a face is presented in competition with another similarly salient stimulus (i.e., another face), visual awareness may be required for it to be processed sufficiently. As a result, divergence in the N170 (130–190 ms) between fearful and neutral faces was not found prior to the emergence of visual awareness (i.e., the VAN; 200–300 ms).

Interestingly, in the later P3 time window (400–500 ms), the ERPs were increased for fearful compared to neutral faces, however, only when stimuli were presented supraliminally and when they were attended. It thus appears that, unlike the earlier processing stage indexed by the N2, the later post-perceptual evaluation of emotional stimuli, indexed by the P3, needs both spatial attention and awareness. This finding is consistent with the view that attentional focus is necessary to elicit emotion-related enhancement on the ERPs during later stages of visual processing [2,57]. The sensitivity of the P3 to attentional control has been suggested to reflect capacity limitations of the neural system. Specifically, because attentional resources are limited, an elaborate evaluation of the stimuli can only operate when the stimuli are attended. Moreover, functionally, the enhanced P3 for spatially attended stimuli may reflect stronger information encoding and consolidation in working memory [58] and enhanced neural representation of stimuli of motivational significance [57], which are necessary for correctly performing the task.

In conclusion, in a paradigm using a bilateral presentation of fearful and neutral human faces, we found that spatial attention focus is not necessary to elicit perceptual awareness, as indexed by the VAN, but is able to enhance it. In addition, while visual awareness is necessary for the processing of emotional faces during both early and late stages of processing (i.e., N2 and P3), spatial attention focus is required for emotion processing only at the later stage (i.e., P3).

## Data Availability

The data presented in this study can be accessed via https://osf.io/zm4qp/ (accessed on 19 May 2022).
